# Supplementary figures and images for: Electronic recording of lifetime locomotory activity patterns of adult medflies
Source: PLoS One. 2022 Jul 25;17(7):e0269940. doi: 10.1371/journal.pone.0269940 (PMC9312368; doi:10.1371/journal.pone.0269940)

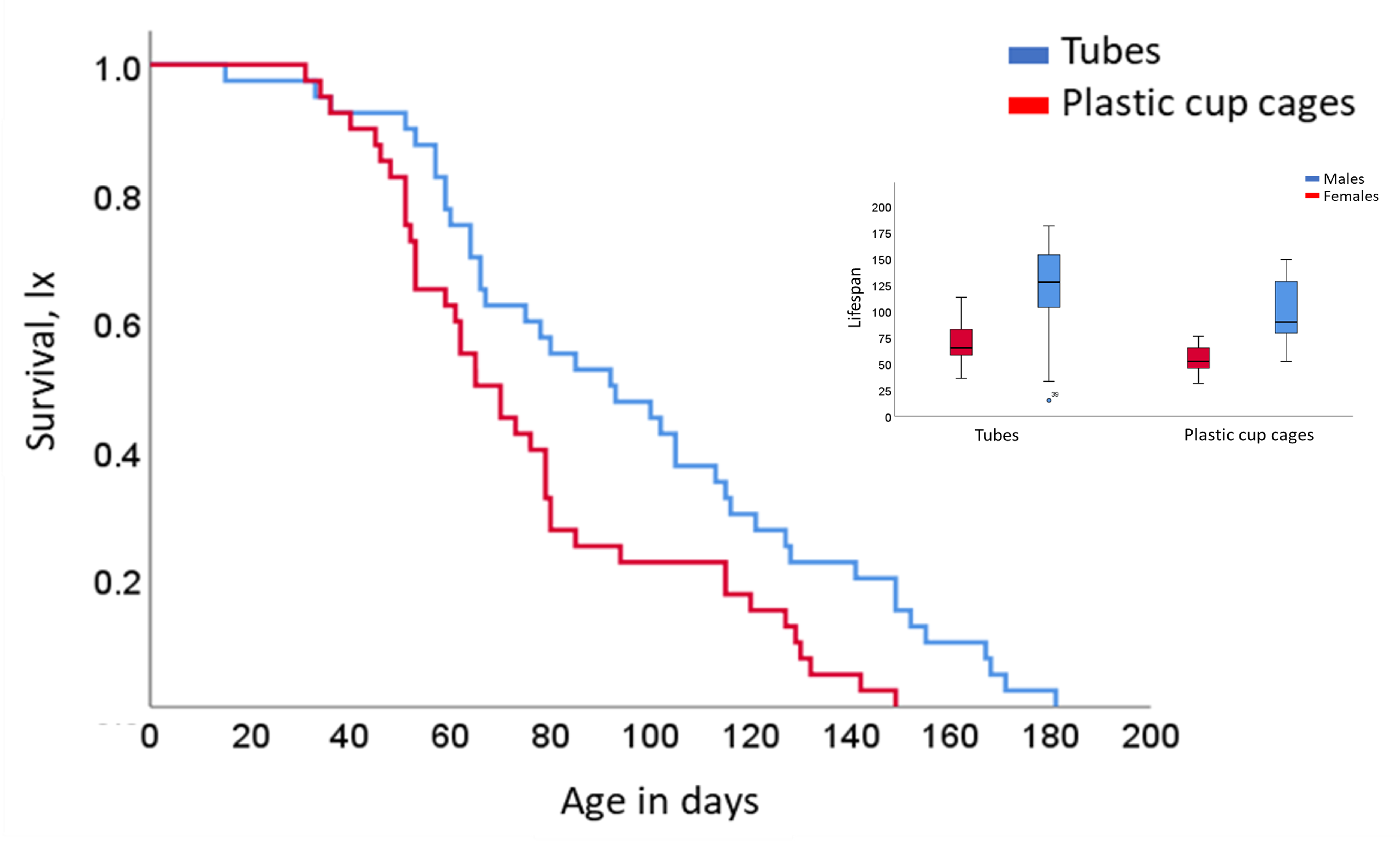

Supplement: S1 Fig — (PNG) [file pone.0269940.s001.png]
