# Supplementary material for: Electronic recording of lifetime locomotory activity patterns of adult medflies
Source: PLoS One. 2022 Jul 25;17(7):e0269940. doi: 10.1371/journal.pone.0269940 (PMC9312368; doi:10.1371/journal.pone.0269940)
Supplement: S1 Table — (DOCX) [file pone.0269940.s002.docx]

# **Supporting information**

**S1 Table:**

| **Factor** | **IRR** | ***P* value** |
| --- | --- | --- |
| **2** | | |
| **Sex (ref: female)** | 0.845 | 0.099 |
| **Night / Morning** | 0.114 | < 0.001 * |
| **Night / Midday** | 0.255 | < 0.001 * |
| **Night / Evening** | 0.237 | < 0.001 * |
| **Morning / Midday** | 2.240 | < 0.001 * |
| **Morning / Evening** | 2.083 | < 0.001 * |
| **Midday / Evening** | 0.930 | 0.645 |
| **11** | | |
| **Sex (ref: female)** | 0.675 | < 0.001 * |
| **Night / Morning** | 0.159 | < 0.001 * |
| **Night / Midday** | 0.307 | < 0.001 * |
| **Night / Evening** | 0.401 | < 0.001 * |
| **Morning / Midday** | 1.923 | < 0.001 * |
| **Morning / Evening** | 2.514 | < 0.001 * |
| **Midday / Evening** | 1.307 | 0,002 * |
| **21** | | |
| **Sex (ref: female)** | 0.668 | < 0.001 * |
| **Night / Morning** | 0.168 | < 0.001 * |
| **Night / Midday** | 0.232 | < 0.001 * |
| **Night / Evening** | 0.349 | < 0.001 * |
| **Morning / Midday** | 1.382 | 0.0012 * |
| **Morning / Evening** | 2.082 | < 0.001 * |
| **Midday / Evening** | 1.507 | < 0.001 * |
| **42** | | |
| **Sex (ref: female)** | 0.493 | < 0.001 * |
| **Night / Morning** | 0.145 | < 0.001 * |
| **Night / Midday** | 0.226 | < 0.001 * |
| **Night / Evening** | 0.278 | < 0.001 * |
| **Morning / Midday** | 1.557 | < 0.001 * |
| **Morning / Evening** | 1.914 | < 0.001 * |
| **Midday / Evening** | 1.230 | 0.124 |
| **81** | | |
| **Sex (ref: female)** | 0.481 | 0.052 |
| **Night / Morning** | 0.170 | < 0.001 * |
| **Night / Midday** | 0.350 | < 0.001 * |
| **Night / Evening** | 0.660 | 0.339 |
| **Morning / Midday** | 2.050 | < 0.001 * |
| **Morning / Evening** | 3.870 | < 0.001 * |
| **Midday / Evening** | 1.890 | 0.0022 * |
