# Supplementary material for: Electronic recording of lifetime locomotory activity patterns of adult medflies
Source: PLoS One. 2022 Jul 25;17(7):e0269940. doi: 10.1371/journal.pone.0269940 (PMC9312368; doi:10.1371/journal.pone.0269940)
Supplement: S2 Table — (DOCX) [file pone.0269940.s003.docx]

| Treatment | eggs/ female ± SE | Mean duration of reproduction periods  in days ± SE | | |
| --- | --- | --- | --- | --- |
|  |  | Pre- oviposition period | Oviposition period | Post oviposition period |
| Tubes | 946,13 ± 70,97 a | 5,24 ± 0,6 a | 64,87 ± 4,86 a | 3,31 ± 0,77 a |
| Plastic cup cages | 624,7 ± 55,57 b | 10,15 ± 0,84 b | 38,6 ± 3,17 b | 5,75 ± 2,3 a |
