# Supplementary material for: Electronic recording of lifetime locomotory activity patterns of adult medflies
Source: PLoS One. 2022 Jul 25;17(7):e0269940. doi: 10.1371/journal.pone.0269940 (PMC9312368; doi:10.1371/journal.pone.0269940)
Supplement: Attachment — Submitted filename: PONE-D-22-00246-annotated.pdf [file pone.0269940.s004.pdf]

# PLOS ONE

## Electronic recording of lifetime locomotory activity patterns of adult medflies

--Manuscript Draft--

| | |
|---|---|
| **Manuscript Number:** | PONE-D-22-00246 |
| **Article Type:** | Research Article |
| **Full Title:** | Electronic recording of lifetime locomotory activity patterns of adult medflies |
| **Short Title:** | Lifetime activity patterns of adult medflies |
| **Corresponding Author:** | Nikos T Papadopoulos<br>University of Thessaly School of Agricultural Sciences<br>Volos, Magnesia GREECE |
| **Keywords:** | |
| **Abstract:** | Age specific and diurnal patterns of locomotory activity, that can be considered as biomarkers of aging in model organisms, vary across the lifetime of individuals. The Mediterranean fruit fly (medfly), Ceratitis capitata , is a commonly used model-species in studies regarding demography and aging. In the present study, we introduce a modification of the automated locomotory activity electronic device LAM25system (Locomotory Activity Monitor) – Trikinetics, commonly used in short time studies, to record the daily locomotory activity patterns of adult medflies throughout the life. Fecundity rates and survival of adult medflies were recorded as well. Male and female medflies were kept in the system tubes and had access to an agar-based gel diet, which apart from nutrients also provided water. The locomotory activity was recorded at every minute by three monitors of the electronic device. The locomotory activity of females was higher than that of males across the different ages. For both sexes locomotory rates were high during the first 20 days of the adult life and decreased in older ages. The activity of males was high in the morning and late afternoon hours, while that of females rather constantly high throughout the photophase. Negligible locomotory activity was recorded for both sexes during the nighttime. Males outlived females. Fecundity of females was higher in younger ages. Our results support the adoption of LAM25system in studies addressing the healthy aging of organisms using medfly as a model organism. |
| **Order of Authors:** | Vasilis G. Rodovitis |
| | Stella A. Papanastasiou |
| | Evmorfia P. Bataka |
| | Christos T. T. Nakas |
| | Nikos A. Koulousis |
| | James R. Carey |
| | Nikos T. T. Papadopoulos |
| **Additional Information:** | |
| **Question** | **Response** |
| **Financial Disclosure**<br><br>Enter a financial disclosure statement that describes the sources of funding for the work included in this submission. Review the submission guidelines for detailed requirements. View published research articles from *PLOS ONE* for specific examples. | Research funded in part by a grant from the Center for the Economics and Demography of Aging, UC Berkeley (NIH 2P30AG012839) |

*Powered by Editorial Manager® and ProduXion Manager® from Aries Systems Corporation*

| | |
|---|---|
| This statement is required for submission and **will appear in the published article** if the submission is accepted. Please make sure it is accurate.<br><br>**Unfunded studies**<br>Enter: *The author(s) received no specific funding for this work.*<br><br>**Funded studies**<br>Enter a statement with the following details:<br>• Initials of the authors who received each award<br>• Grant numbers awarded to each author<br>• The full name of each funder<br>• URL of each funder website<br>• Did the sponsors or funders play any role in the study design, data collection and analysis, decision to publish, or preparation of the manuscript?<br>• **NO** - Include this sentence at the end of your statement: *The funders had no role in study design, data collection and analysis, decision to publish, or preparation of the manuscript.*<br>• **YES** - Specify the role(s) played.<br><br>* typeset | |
| **Competing Interests**<br><br>Use the instructions below to enter a competing interest statement for this submission. On behalf of all authors, disclose any competing interests that could be perceived to bias this work—acknowledging all financial support and any other relevant financial or non-financial competing interests.<br><br>This statement is required for submission and **will appear in the published article** if the submission is accepted. Please make sure it is accurate and that any funding sources listed in your Funding Information later in the submission form are also declared in your Financial Disclosure statement.<br><br>View published research articles from *PLOS ONE* for specific examples. | The authors have declared that no competing interests exist. |

| | |
|---|---|
| **NO authors have competing interests**<br><br>Enter: *The authors have declared that no competing interests exist.*<br><br>**Authors with competing interests**<br><br>Enter competing interest details beginning with this statement:<br><br>*I have read the journal's policy and the authors of this manuscript have the following competing interests: [insert competing interests here]*<br><br>* typeset | |
| **Ethics Statement**<br><br>Enter an ethics statement for this submission. This statement is required if the study involved:<br><br>• Human participants<br>• Human specimens or tissue<br>• Vertebrate animals or cephalopods<br>• Vertebrate embryos or tissues<br>• Field research<br><br>Write "N/A" if the submission does not require an ethics statement.<br><br>General guidance is provided below. Consult the submission guidelines for detailed instructions. **Make sure that all information entered here is included in the Methods section of the manuscript.** | N/A |

| | |
|---|---|
| **Format for specific study types**<br><br>**Human Subject Research (involving human participants and/or tissue)**<br>• Give the name of the institutional review board or ethics committee that approved the study<br>• Include the approval number and/or a statement indicating approval of this research<br>• Indicate the form of consent obtained (written/oral) or the reason that consent was not obtained (e.g. the data were analyzed anonymously)<br><br>**Animal Research (involving vertebrate animals, embryos or tissues)**<br>• Provide the name of the Institutional Animal Care and Use Committee (IACUC) or other relevant ethics board that reviewed the study protocol, and indicate whether they approved this research or granted a formal waiver of ethical approval<br>• Include an approval number if one was obtained<br>• If the study involved *non-human primates*, add *additional details* about animal welfare and steps taken to ameliorate suffering<br>• If anesthesia, euthanasia, or any kind of animal sacrifice is part of the study, include briefly which substances and/or methods were applied<br><br>**Field Research**<br><br>Include the following details if this study involves the collection of plant, animal, or other materials from a natural setting:<br>• Field permit number<br>• Name of the institution or relevant body that granted permission | |
| **Data Availability**<br><br>Authors are required to make all data underlying the findings described fully available, without restriction, and from the time of publication. PLOS allows rare exceptions to address legal and ethical concerns. See the PLOS Data Policy and FAQ for detailed information. | Yes - all data are fully available without restriction |

| | |
|---|---|
| A Data Availability Statement describing where the data can be found is required at submission. Your answers to this question constitute the Data Availability Statement and **will be published in the article**, if accepted.<br><br>**Important:** Stating 'data available on request from the author' is not sufficient. If your data are only available upon request, select 'No' for the first question and explain your exceptional situation in the text box.<br><br>Do the authors confirm that all data underlying the findings described in their manuscript are fully available without restriction? | |
| **Describe where the data may be found in full sentences. If you are copying our sample text, replace any instances of XXX with the appropriate details.**<br><br>• If the data are **held or will be held in a public repository**, include URLs, accession numbers or DOIs. If this information will only be available after acceptance, indicate this by ticking the box below. For example: *All XXX files are available from the XXX database (accession number(s) XXX, XXX.*).<br>• If the data are all contained **within the manuscript and/or Supporting Information files**, enter the following: *All relevant data are within the manuscript and its Supporting Information files.*<br>• If neither of these applies but you are able to provide **details of access elsewhere**, with or without limitations, please do so. For example:<br><br>*Data cannot be shared publicly because of [XXX]. Data are available from the XXX Institutional Data Access / Ethics Committee (contact via XXX) for researchers who meet the criteria for access to confidential data.*<br><br>*The data underlying the results presented in the study are available from (include the name of the third party* | https://data.world/vasrod43/lam25system-medfly2021 |

| | |
|---|---|
| *and contact information or URL).*<br>• This text is appropriate if the data are owned by a third party and authors do not have permission to share the data.<br><br>* typeset | |
| Additional data availability information: | Tick here if the URLs/accession numbers/DOIs will be available only after acceptance of the manuscript for publication so that we can ensure their inclusion before publication. |

Powered by Editorial Manager® and ProduXion Manager® from Aries Systems Corporation

Manuscript Click here to access/download;Manuscript;Manuscript.docx

# Electronic recording of lifetime locomotory activity patterns of adult medflies

**Vasilis G. Rodovitis[1], Stella A. Papanastasiou[1], Evmorfia P. Bataka[2], Christos T. Nakas[2,6] Nikos A. Koulousis[3], James R. Carey[4,5] and Nikos T. Papadopoulos[1*]**

[1] Laboratory of Entomology and Agricultural Zoology, Department of Agriculture, Crop Production and Rural Environment, University of Thessaly, Volos, Greece

[2] Laboratory of Biometry, Department of Agriculture, Crop Production and Rural Environment, University of Thessaly, Volos, Greece

[3] Laboratory of Applied Zoology and Parasitology, School of Agriculture, Aristotle University of Thessaloniki, Thessaloniki, Greece

[4] Department of Entomology University of California Davis, California USA

[5] Center for the Economics and Demography of Aging University of California Berkeley, California USA

[6] University Institute of Clinical Chemistry, Inselspital, Bern University Hospital, University of Bern, Bern, Switzerland

[*] Correspondence to: nikopap@uth.gr

# 1. Abstract

Age specific and diurnal patterns of locomotory activity, that can be considered as biomarkers of aging in model organisms, vary across the lifetime of individuals. The Mediterranean fruit fly (medfly), *Ceratitis capitata*, is a commonly used model-species in studies regarding demography and aging. In the present study, we introduce a modification of the automated locomotory activity electronic device LAM25system (Locomotory Activity Monitor) – Trikinetics, commonly used in short time studies, to record the daily locomotory activity patterns of adult medflies throughout the life. Fecundity rates and survival of adult medflies were recorded as well. Male and female medflies were kept in the system tubes and had access to an agar-based gel diet, which apart from nutrients also provided water. The locomotory activity was recorded at every minute by three monitors of the electronic device. The locomotory activity of females was higher than that of males across the different ages. For both sexes locomotory rates were high during the first 20 days of the adult life and decreased in older ages. The activity of males was high in the morning and late afternoon hours, while that of females rather constantly high throughout the photophase. Negligible locomotory activity was recorded for both sexes during the nighttime. Males outlived females. Fecundity of females was higher in younger ages. Our results support the adoption of LAM25system in studies addressing the healthy aging of organisms using medfly as a model organism.

# 2. Introduction

Aging is the gradual decline in physiological integrity and function combined with increased chance of death and reduced probability to reproduce [1,2]. “Normal” aging is defined as the summary of physiological changes that occur in an organism over time [3], while “functional senescence” describes the progressive decline in activity and function with aging [4]. Age-related behavioral changes result in progressive deterioration of many spontaneous responses including locomotory activity [3]. The spontaneous behavior of organisms is directly associated with movement and catalyzes the ability of the individual to respond to environmental stimuli and to daily vital needs, such us survival and reproduction, which are firmly correlated with its fitness traits and age [5].

Model organisms are non-human species which are used in biological research to investigate, among others, complex behaviors and biological phenomena and to provide information regarding life history traits of humans and mammals [6, 7]. Fruit flies’ activity ~~and several behaviors~~ vary during the life course of the individuals, while some of them may clearly decline or increase with age, hence can be exploited in demographic, aging and behavioral research [8-11]. Changes in spontaneous behavior and locomotory activity in relation to age had been described in *Drosophila* species and the Mediterranean fruit fly providing insights to understand the aging process [8, 11- 15].

In this study, we used the Mediterranean fruit fly (medfly), *Ceratitis capitata* (Wiedemann) (Diptera: Tephritidae), as a model organism to study age related patterns of locomotory activity. Medfly is commonly used as a model organism in demographic and gerontology studies [11, 14, 16-18]. ~~Previous studies showed that the daily frequency of supine behavior was positively correlated with the time of the individual’s death, while the frequency of performing sexual signaling by the males was negatively correlated with the predicted time to death [17, 19, 20].~~

Differences of daily behavioral patterns in adult medflies were previously attributed to sex and age. Males are in general more active and longer-lived than female[illegible]nd sexual signaling represents their foremost activity during the day [21]. Generally, a voluminous part of literature examines the age-related patterns of Drosophilids’ and Tephritids’ behavior [3, 4, 11] but only few of them examine the details of locomotory activity throughout the lifespan of individuals[illegible]e combination of lifetime locomotory activity with survival and reproduction is expected to generate important data towards understanding functional senescence in model organisms.

Ranging from simple visual observation to the use of various electronic devices [5], spontaneous activity and behavior of flies have been investigated in the past. The simplest approach is the “grid of square” method [22], where flies are kept individually in a chamber with a grid of circles drawn on its base. The number of crossed circles by the adult define the level of activity [22]. Ewing [23] developed the “labyrinth” method, where a series of cones allowed a unidirectional passage of individuals. Flies were released in the first cone-compartment and the distribution of them in the next compartments, generated data to assess the level of activity. Devices such as the “Actimeter” and the “Locomotron”, consisting of a small chamber with an infrared light gate located in the center, are examples of automated equipment for measuring activity. An activity event is recorded whenever the

fly crosses the light gate. Several variants of these apparatus have been developed, depending on the purpose of the study. Automated devices based on video-camera tracking systems have been greatly improved in the last decade and were used to record the activity of fruit flies and *Drosophila* species [14, 24-26]. For example, Zou et al. [14] and Chiu et al. [27], using a Behavioral Monitoring System- BMS, recorded the lifetime daily behavior in females of *Anastrepha ludens*, in relation to food quality, revealing different diurnal patterns of behavior of young, middle-aged, and old adults. The need for constant monitoring individual insects, under fluctuating laboratory conditions and for long time periods, led to the development of high quality, automated devices to tract their movement [28]. The LAMsystem- Locomotory Activity Monitor and DAMsystem- Drosophila Activity Monitor (Trikinetics), are extensively used devices for this type of assays [28-30].

The effect of different environmental and rearing conditions on the locomotory activity of fruit flies was traditionally measured by visual observations, which are time consuming, and observer biased. The introduction, during the last two decades, of the automatic DAM and LAM systems simplified the study of locomotory activity of fruit flies. For example, using the LAM system (in the frameworks of quality control tests of Sterile Insect Technique programs), the daily locomotory activity of *Bactrocera tryoni* adults were found to be positively correlated with protein and nutrient concentration in adult diet [26, 31]. Utilizing the same system, Dominiak et al. [32] defined the effect of sterilization on the activity of adult *B. tryoni*, while Weldon et al. [24], using an artificial video system, reported the differences in daily behavior between fertile and sterile adults. The LAM system was recently used to determining differences in the activity patterns of *B. tryoni* adults with or without access to a diet rich in endosymbiotic bacteria [33], and of a wild-type and a self-limiting transgenic strain of *Bactrocera oleae* population [34].

Day and night lifetime locomotory activity has not been previously assessed in *C. capitata* adults. The aim of the present study was to establish a system to automatically record the activity and reproduction of adult fruit flies throughout their lifespan as a foundation for research in aging and health dynamics using invertebrate model systems. For this purpose, we used, for the first time, the LAM25system by Trikinetics which records the movement of the flies on three different spots within the chamber where they were individually kept. We also recorded the fecundity of females through daily visual observations. ~~The methods and results of this study form a baseline dataset which could pave the way for new research dealing with fundamental questions of gerontology and aging of flies.~~

# 3. Materials and Methods

## 3.1. Flies and rearing conditions

The study was conducted in the Laboratory of Entomology and Agricultural Zoology at the University of Thessaly, Greece, from September 2019 to December 2020, under constant laboratory conditions, 25 ± 2 $^{o}$C, 65 ± 5 % RH, and a photoperiod of L14:D10 with photophase starting at 7:00 am and ending at 9:00 pm[illegible]e used males and females of the Mediterranean fruit fly laboratory strain “Benakio”, that has been maintained under laboratory conditions for more than 30 years [35].

The rearing procedure of flies was implemented as described in [36] and [37], by keeping adults in groups of 100 individuals in wooden, wire-screened cages (30 × 30 × 30 cm) provided, *ad libitum*, with water and an adult diet consisting of a mixture of yeast hydrolysate, sugar, and water at a 4:1:5 ratio. Females deposited eggs on the inner surface of an artificial substrate (dome), comprised by a hollow, red plastic hemisphere (5 cm Ø), which was punctured with 40–50 evenly distributed holes (1 mm Ø). Each dome was fitted in the cover of a plastic Petri dish (5.5-cm Ø). A plastic cup with 0.5 ml of orange juice was added in every Petri dish to stimulate oviposition. Water was placed in the Petri dish to maintain high humidity levels in the dome for female oviposition [38]. Domes were placed in rearing cages for 24h to collect eggs for the experimental needs. Collected eggs were placed on a cotton disk[illegible]ving as a bulking agent for larval diet, within a petri dish. We placed 100 eggs per cotton disk which was previously soaked in the larval diet consisting of 200 g sugar, 200 g brewer’s yeast, 100 g soybean flour, 4 g salt mixture, 16 g ascorbic acid, 16 g citric acid, 3 g sodium propionate, and 1 l water [38]. Petri dishes were placed into plastic containers on a layer of sterilized sand, where larvae pupated.

## 3.2. Assaying locomotory activity patterns of adult medflies

We recorded the locomotory activity patterns of adult medflies by using the Locomotory Activity Monitor- LAM25system (Trikinetics). In this system, flies were individually kept in 32 glass tubes (25 mm Ø, 125 mm length). Each tube was vertically crossed by three ray-rings of infrared light beams (monitors) at three different planes (close to the two ends of the tube and one in the middle) (Fig 1A). Activity was measured every minute, as the number of times the fly passed through an infrared beam.

The day that adults emerged, fifteen males and sixteen females were individually positioned in the tubes and kept throughout in there until death. Maintenance of flies in the tubes was ensured with minimum frequency of disturbances (jolts, flicks, shakes) that would affect their normal activity. We prepared an agar-based gel diet consisting of sugar, yeast

hydrolysate, agar, nipagin and water (4:1:0.2:0.1:20) to provide adults with both nutrients and water simultaneously. The gel diet was replaced every four days to avoid dehydration.

On the one end of the tube, we adjusted a vinyl plastic stopper (CAP25-BLK- Vinyl Tube Cap- Trikinetics) with the gel diet and on the other end (Fig 1B), a cap with organdie cloth (Fig 1C), which served for the ventilation of tubes and also as an oviposition substrate (Fig 1D) [39]. Outside the oviposition substrate end of the tube, we adjusted a wooden, custom-made frame with four Plexiglas platforms (Fig 1E), where the eggs deposited by each female landed on a black filter paper. Adult mortality and the number of deposited eggs were daily recorded with visual observations until the death of all individuals.

**Fig. 1.:** Side view of the LAM25system set in the laboratory (A). Vinyl plastic stopper placed on one end with the agar-based gel diet (B). Cap with organdie cloth placed on the other end for ventilation of tubes (C) and for egg deposition (D). Wooden- plastic, handmade construction, for egg collection (E).

## 3.3. Data Processing and Analyses

We calculated the mean daily (24h) locomotory activity of every male and female, recorded in one-minute intervals throughout their lifetime. The LAM25system was set to record, in each of the three monitors, the sum of movements an adult performed every minute. To calculate the mean activity of an individual for a given period of time, we summarized the recordings of the three monitors per minute during this period. Then, we summarized the recordings for every individual during 24h (1440 recordings), during daytime (840 recordings) and during nighttime (600 recordings). Accordingly, we constructed three event-history diagrams (24h interval, daytime – 14h interval, and night – 10h interval). An event-history diagram [40], depicts age-specific activity patterns using different color-coding for different levels of activity. Adult flies are represented in the diagram by horizontal "lines" proportional to their lifespan. Activity is color-grouped according to the summarized movements of an adult during the tested period. Three activity levels are represented by a separate color (low-green, medium-yellow, high-red).

We also calculated the mean activity per 24h interval for the cohorts of males and females, recorded during four selected ages (2, 11, 21, 42 and 81 days old). More specifically, for every individual, we summarized the recordings of the three monitors per minute, during the 24h interval for each selected day. The five selected ages were characterized as: newly emerged adults (2-days old), reproductively mature adults (11-days old), middle aged adults (21-days old), elderly adults (42-days old) and extreme aged adults (81 days old) [6, 41].

Survival analysis for males and females during their maintenance in the tubes of LAM25system was conducted with IBM SPSS 26.0 (IBM Corp., Armonk, NY, U.S.A.). Differences in survival between the two sexes were tested with the Cox proportional hazards model.

Activity data were analyzed with R v.4.0.0 (R Foundation for Statistical Computing, Vienna, Austria) using the “geepack” package for Generalized Estimating Equations (GEEs) [42] to account for within-subject dependency. Clusters of subjects that were considered within subject observations are correlated (time-dependency). The Poisson model was used for the response variable “Activity” since the response variable represents count data (sum activity/ fly/day). The effect of age and sex on average daily activity of flies was also tested. The average daily activity was estimated by the sum of daily movements recorded on the three monitors for each fly per minute, divided by the total number of flies which survived each day until the age of 91 days. Finally, the total activity of flies per time - period was compared according to two different factors a) sex and b) time period during the 24hours of day: night (21:00- 6:59), morning (7:00 – 13:59), midday (14:00 – 17:59), evening (18:00 – 20:59) for 5 different ages (2, 11, 21, 42, 81 days old). Parameter estimates are presented as Incidence Rate Ratios (IRR) with 95% confidence intervals (CI), which is the ratio of the activity level in a group of interest to the number of activities of the group used as reference. To interpret the results, for IRR >1, the activity is higher for the group of interest rather than the reference group, while IRRs lower than 1 indicate less activity for the group of interest comparing to the reference. Pairwise contrasts for estimated marginal means were assessed using the Tukey adjustment for multiple comparisons.

# 4. Results

## 4.1. Demographic parameters

Males lived longer than females (Wald $\chi^2 = 5.38$, df = 1, $P = 0.02$) (Fig 2). The first dead adult was observed on day 48 and on day 53 of age, for males and females, respectively. Female mortality rates increased dramatically after the age of 50 days compared to males. Half of the female and male cohorts survived 59 and 74 days, respectively (Fig 2A). The lifespan of the longest-lived male (166 days) differed by 75 days from that of the longest-lived female (91 days).

**Fig. 2:** Age-specific survival curves with 95% Confidence Intervals (CI) (A) and box plots depicting lifespan of males and females kept in the tubes of the LAM25system.

The mean lifetime fecundity was 973.4 ± 56.6 (SE) eggs per femal[illegible]males reached their maximum oviposition rate on the 13th day of age with an average of 42.6 ± 4.6 eggs (Fig 3). Fecundity rates followed a linear downward trend from day 30 of age onwards.

**Fig. 3:** LOESS curves (Locally Estimated Scatterplot Smoothing curves) for age specific fecundity rates with 95% Confidence Intervals (CI) of females kept in the tubes of LAM25system.

## 4.2. Activity

### Age-specific locomotory activity

The age specific locomotory activity patterns of males and females is ~~given~~ in Fig 4. Activity levels were estimated for the whole 24h interval, as well as for daytime and nighttime separately. Females were more active than males during the 24h and the daytime periods regardless of their age (Table 1; Fig 4). The locomotion activity of both sexes declined with age and was not affected by the tested period (24h, daytime, nighttime). No significant differences were recorded in activity levels between the two sexes during nighttime (Table 1). The pattern of declining activity with aging of males both during day- and nighttime was smooth and progressive. Age specific patterns in female activity were high during the first 20 days of adult life, declining sharply to day 30 stabilized up to approximately day 45 and then declining again to day 60 and stabilized to day 80 of age. Age specific patterns of females differed a great deal between day and night.

**Table 1**: **Variables of the GEEs with significant effects on the age-specific activity of flies during the 24h day-night interval, the daytime and the nighttime periods, for their entire lifespan, by the factors: sex (males, females) and age**.

| | Day and night | | Day | | Night | |
|---|---|---|---|---|---|---|
| **Factor** | **IRR (95% CI)** | ***P* value** | **IRR (95% CI)** | ***P* value** | **IRR (95% CI)** | ***P* value** |
| **Sex (ref: female)** | 0.710 (0.623, 0.809) | < 0.001 * | 0.687 (0.605, 0.782) | < 0,001 * | 0.858 (0.698, 1.050) | 0.144 |
| **Age** | 0.986 (0.983, 0.988) | < 0.001 * | 0.985 (0.983, 0.987) | < 0,001 * | 0.989 (0.987, 0.992) | < 0,001 * |

**Fig. 4:** LOESS curves (Locally Estimated Scatterplot Smoothing curves) for mean activity of males (blue) and females (red) with 95% Confidence Intervals (CI), during the 24h period (A), daytime (B) and nighttime (C). Mean activity was estimated by the sum of movements of all individuals during the period of interest (24h, daytime, nighttime), divided by the number of individuals surviving on each day.

An event history chart was used to depict the detail of age-specific locomotory activity of males and females for the whole 24h period, as well as for daytime and nighttime periods (Fig 5). The females were more active than males throughout their lifespan. Males exhibit high levels of locomotory activity (>2000 movements) during the first 10 days of their life. Activity levels remained at a rather medium level (501-2000) from day 10 to day 60 and further reduced in older ages. The activity of females was high for all individuals during the first 20 days. High variability in activity patterns among females was reported from day 30 to 60. During nighttime, no different patterns in age-specific locomotory activity were observed between the two sexes. All flies were moribund for one or two days before death.

**Fig. 5:** Event history diagram providing a detailed picture of the ages when activity was recorded during the 24h interval (A), daytime (B) and nighttime (C). Red: > 2000 movements during 24h and daytime and > 200 during nighttime, yellow: 501-2000 movements during 24h and daytime and 51-200 during nighttime, green: 0-500 movements during 24h and daytime and 0-50 during nighttime.

## Fine- scale 24h locomotory activity rhythms

Both males and females were more active during daytime (from the beginning until the end of the photophase) and remained almost motionless during darkness, regardless of the age group (Fig 6). Locomotory activity started at approximately 06:00, shortly before the start of the photophase (7:00) and stopped at approximately 22:00 shortly after its end (21:00), lasting for almost 16 hours. Because flies of the different age groups exhibit different patterns of locomotory activity we separate the 24h interval in four different periods (night, morning, midday and evening) to compare males and females (Table B in S1 Text). Male medflies exhibit a specific diurnal pattern of sexual signaling characterized by increased intensity in the morning and afternoon hours as it has been demonstrated earlier [8, 19, 36].

No significant differences were recorded in the activity patterns between males and females on the 2$^{nd}$ and 81$^{st}$ day of life, adjusting for day period (Table B in S1 Text). During the photophase, flies were more active in the morning and less active in the midday and the evening. Reproductively mature (11-days old), middle aged (21-days old) and elderly (42-days old) females were more active than males, adjusting for day period. Flies of the three respective ages were more active during the morning than in the midday and evening. The lowest activity was recorded during the night for all five ages tested (Table B in S1 Text).

**Fig. 6:** LOESS curves (Locally Estimated Scatterplot Smoothing curves) with 95% Confidence intervals (CI) of the mean 24h locomotory activity for males and females at ages 2, 11, 21, 42 and 81 days.

# 5. Discussion

This is the first study on the fine-scale patterns of lifetime locomotory activity for adult medflies. A major advance of our study includes the adaptation of the LAM25system for long-term activity recordings (almost six months), as until now this system has been only used for short time studies (few days). For example, the LAMsystem has been used to record activity levels of newly emerged adult fruit flies, mainly addressing quality control issues, for their use in SIT programs [24, 26, 31, 32]. Also, the same system has been implemented to characterize the circadian rhythms and sleep patterns of other Diptera species such as Drosophilids but only for a few days [28, 29, 43]. However, Koh and co-workers [12], measured lifetime activity of *D. melanogaster* adults using the DAMsystem, but flies had to be transferred often to new vials to have access to fresh food. Apart from extending the study of locomotory activity to the whole lifespan of the tested organism, we managed to simultaneously record reproduction. For this purpose, we developed and adjusted an egg laying system to the LAM25system, while flies were kept in good health in the tubes. Our approach enabled the recording of adult survival and female fecundity daily, without causing disturbances in the system that would affect the activity of the flies. The current modified system can be used to answer questions regarding (a) the cost of reproduction and effects of activity on adult survival, (b) the aging process, and (c) health dynamics using medfly as a model organism.

In agreement with previous studies, our results demonstrate that males outlived females [44, 46-48]. Females facing higher reproductive cost, due to the reproductive system attrition caused by egg production and oviposition events, which leads on decreased longevity. Females medflies exhausting their reproductive potential rapidly, are predicted to have shorter lifespan [41]. However, in males the higher rates of sexual signaling in young ages is strongly correlated with higher lifespan [49]. Apparently, males face a lower cost of reproduction than females [8, 21]. In our study, young females (9th-17th day) laid more eggs, while from the 35th day onwards the fecundity rates substantially decreased, and the lowest egg production was recorded in older females. According to the models of Müller et al. [41], and Novoseltsev et al. [50], after attaining peak rates the fecundity of female medflies decreases rapidly, and the mortality gradually supervenes. Our results are alinged with both studies above since mortality rates of females increased rapidly after day 50 and daily egg laying dramatically decorate after peak was recorded. Although young female medflies invest high levels of energy in egg production and older ones in survival [18], this strategy is not sufficient to prolong lifespan in comparable levels to that of males.

Age specific locomotory activity showed a declining trend with age in both sexes. Females were more active than males throughout lifespan, in contrast to the expression of other specific behaviors of medfly [21]. In other tephritid species, such as *B. tryoni* [31] males were more active than females. Medfly males were found to perform sexual signaling with greater frequency and duration throughout most of the photoperiod [8, 19, 21]. Apparently, males performing sexual signaling remain stationary and hence no activity/ movement can be recorded by the LAM25system. Sexual signaling is an indicator of longevity, and the frequency of performing it is positively correlated with males' survival [19, 21, 51, 52]. The LAM25system can only detect movement and therefore could not record the

sexual signaling behavior of males. Although not directly measured, we can assume that differences in age specific locomotory activity between males and females resulted mainly because males invested most of the time during photophase in performing sexual signaling.

Locomotory and fecundity rates of female medflies were high at young ages (first 20 days) and then gradually declined. A progressive decrease in daily activity with increasing age has been previously reported in female medflies [11, 17, 19, 21], as well as in both sexes of *A. ludens* [14] and of *D. melanogaster* [3, 4, 53]. Walking, flying, and feeding frequencies were higher at young ages of medfly and *D. melanogaster*, in contrast to other motionless behaviors like resting and grooming, which remained in similar levels despite aging [11]. In agreement with our results, daily activity patterns of *Drospophila* spp. differed by sex, age and reproductive maturity [5, 12, 54] with young flies being more active and fecund than older ones. According to Bochicchio et al. [55], and references therein, aging is directly associated with the gradual reduction of activity and the expression of activity is an indicator of the “functional state” of the individual. Specifically, aging implies a progressive deterioration in the ability to maintain homeostasis, metabolism, and normal functions at high levels [56]. Research concerning the functional deterioration in relation to age identifies key organic systems that fail to function satisfactory in older ages [57]. Both the modification of LAM25system to record lifetime locomotory activity and the results of our study could assist towards understanding the aging process in insect model organisms.

In agreement with previous studies on activity of Tephritidae, our results showed that medfly locomotory activity patterns of selected ages (2, 11, 21, 42, 81 days) were higher during the photoperiod [51], and that activity levels differed between male and females [8, 36, 51, 58-62]. Specifically, both sexes were more active in the morning than in midday and evening. Visual observations confirmed that during evening both sexes invested time in the less active behavior of feeding [51, 58, 59]. During early morning period (7:00- 10:00), males exhibited similar activity with females, but females were more active throughout the rest of the day. Previous studies also showed that early in the morning, males in nature are involved in lekking activities trying to establish or locate leks [51, 58, 59], while during later in the morning and midday they exhibit mainly sexual signaling and remain stationary [8, 58, 36]. A slight increase of activity observed during late evening (on 7:00 pm) could be explained by male re-activation and exhibition of sexual signaling as described earlier by Papadopoulos et al. [8] and Diamantidis et al. [36]. According to Warburg & Yuval [51] and Díaz-Fleischer & Aluja [59], female tephritids perform all type of behaviors throughout the day, without these behaviors being separated by the time period of day, which is in agreement with our findings.

# 6.Conclusion

Our study highlights the modification and use of the LAM25system to record individual lifetime locomotory activity patterns of true fruit flies, in combination with reproduction and survival. Our results revealed a strong connection between decreasing locomotory activity and reproduction with aging. Provided that these factors are strongly correlated with functional senescence of true fruit flies, this system could be exploited in studies of demography and aging process. In addition, our experimental procedure could be used in quality control tests performed in SIT, or in studies dealing with stress physiology and phenotypic plasticity.

Although, the trials reported in the current paper have been conducted with a limited number of individuals, our results succeed to robustly demonstrate several important patterns of activity in medflies, differences between males and females and connection of activity with aging and reproduction. Hence, our study forms the basis for future exploration of the correlation among locomotory activity, functional aging, and reproduction elements.

# 7. References


1. Carey JR, Molleman F. Reproductive aging in tephritid fruit flies. Annals of the New York Academy of Sciences. Blackwell Publishing Inc. 2010; 1204: 139–148.
2. He Y, Jasper H. Studying aging in *Drosophila*. Methods. 2014; 68(1):129–133.
3. Iliadi KG, Boulianne GL. Age-related behavioral changes in *Drosophila*. Annals of the New York Academy of Sciences. Blackwell Publishing Inc. 2010: 1197: 9–18.
4. Simon AF, Liang DT, Krantz DE. Differential decline in behavioral performance of *Drosophila melanogaster* with age. Mech Ageing Dev. 2006; 127(7):647–51.
5. Martin JR. Locomotor activity: A complex behavioural trait to unravel. In: Behavioural Processes. Elsevier. 2003; 145–60.
6. Ankeny RA, Leonelli S. What's so special about model organisms? Stud Hist Philos Sci Part A. 2011; 42(2):313–23.
7. Leonelli S, Ankeny RA. What makes a model organism? Endeavour. 2013; 37(4):209–12.
8. Papadopoulos NT, Katsoyannos BI, Kouloussis NA, Economopoulos AP, Carrey JR. Effect of adult age, food, and time of day on sexual calling incidence of wild and mass-reared *Ceratitis capitata* males. Entomologia Experimentalis et Applicata. 1998; 89(2):175-182.
9. Markowska AL., Breckler SJ. Behavioral biomarkers of aging: illustration of a multivariate approach for detecting age-related behavioral changes. Journals of Gerontology Series A: Biomedical Sciences and Medical Sciences. 1999; 54(12):B549–66.
10. Gargano JW, Martin I, Bhandari P, Grotewiel MS. Rapid iterative negative geotaxis (RING): A new method for assessing age-related locomotor decline in *Drosophila*. Experimental Gerontology. 2005; 40(5):386–95.
11. Carey JR, Papadopoulos N, Kouloussis N, Katsoyannos B, Müller HG, Wang JL, et al. Age-specific and lifetime behavior patterns in *Drosophila melanogaster* and the Mediterranean fruit fly, *Ceratitis capitata*. Exp Gerontol. 2006; 41(1):93–7.
12. Koh K., Evans JM., Hendricks JC., Sehgal A. A *Drosophila* model for age-associated changes in sleep: wake cycles. Proc Natl Acad Sci. 2006; 103(37):13843–7.
13. Jacobson J, Lambert AJ, Portero-Otín M, Pamplona R, Magwere T, Miwa S, et al. Biomarkers of aging in *Drosophila*. Aging Cell. 2010; 9(4):466–77.

14. Zou S, Liedo P, Altamirano-Robles L, Cruz-Enriquez J, Morice A, Ingram DK, et al. Recording lifetime behavior and movement in an invertebrate model. PLoS One. 2011;6(4).

15. Tower J, Agrawal S, Alagappan MP, Bell HS, Demeter M, Havanoor N, et al. Behavioral and molecular markers of death in *Drosophila melanogaster*. Exp Gerontol. 2019; 126, 110707.

16. Novoseltsev VN., Carey J., Liedo P., Novoseltseva J., Yashin AI. Anticipation of oxidative damage decelerates aging in virgin female medflies: hypothesis tested by statistical modeling. Exp Gerontol. 2000; 35(8):971–87.

17. Papadopoulos NT, Carey JR, Katsoyannos BI, Kouloussis NA, Müller HG, Liu X. Supine behaviour predicts the time to death in male Mediterranean fruitflies (*Ceratitis capitata*). Proc R Soc B Biol Sci. 2002; 269(1501):1633–7.

18. Papanastasiou SA, Nakas CT, Carey JR, Papadopoulos NT. Condition-Dependent Effects of Mating on Longevity and Fecundity of Female Medflies: The Interplay between Nutrition and Age of Mating. PLoS One. 2013; 8(7).

19. Papadopoulos NT., Katsoyannos BI., Kouloussis NA., Carey JR., Müller HG., Zhang Y. High sexual signalling rates of young individuals predict extended life span in male Mediterranean fruit flies. Oecologia. 2004; 138(1):127–34.

20. Zhang Y., Müller HG., Carey JR., Papadopoulos NT. Behavioral trajectories as predictors in event history analysis: male calling behavior forecasts medfly longevity. Mech Ageing Dev. 2006; 127(8):680–6.

21. Papanastasiou SA. Effect of aging and of other factors on the behaviour of the Mediterranean fruit fly *Ceratitis capitata* (Diptera: Tephritidae) [Internet]. University of Thessaly; 2014.

22. Connolly K. Locomotor activity in *Drosophila*: II. Selection for active and inactive strains. Anim Behav. 1966; 14:444–9.

23. Ewing AW. Attempts to select for spontaneous activity in *Drosophila melanogaster*. Anim Behav. 1963; 11(2–3):369–78.

24. Weldon CW, Prenter J, Taylor PW. Activity patterns of Queensland fruit flies (*Bactrocera tryoni*) are affected by both mass-rearing and sterilization. Physiol Entomol. 2010; 35(2):148–53.

25. Donelson N, Kim EZ, Slawson JB, Vecsey CG, Huber R, Griffith LC. High-resolution positional tracking for long-term analysis of *Drosophila* sleep and locomotion using the “tracker” program. PLoS One. 2012; 7(5).

26. Prenter J, Weldon CW, Taylor PW. Age-related activity patterns are moderated by diet in Queensland fruit flies *Bactrocera tryoni*. Physiol Entomol. 2013; 38(3):260–7.

27. Chiu JC, Kaub K, Zou S, Liedo P, Altamirano-Robles L, Ingram D, et al. Deleterious effect of suboptimal diet on rest-activity cycle in *Anastrepha ludens* manifests itself with age. Sci Rep. 2013;3(1): 1-9.

28. Rosato E, Kyriacou CP. Analysis of locomotor activity rhythms in *Drosophila*. Nat Protoc. 2006; 1(2):559–68.

29. Pfeiffenberger C, Lear BC, Keegan KP, Allada R. Locomotor activity level monitoring using the Drosophila activity monitoring (DAM) system. Cold Spring Harb Protoc. 2010; 5(11).

30. Pfeiffenberger C, Lear BC, Keegan KP, Allada R. Processing circadian data collected from the Drosophila activity monitoring (DAM) system. Cold Spring Harb Protoc. 2010; 5(11).

31. Fanson BG, Petterson IE, Taylor PW. Diet quality mediates activity patterns in adult Queensland fruit fly (*Bactrocera tryoni*). J Insect Physiol. 2013; 59(7):676–81.

32. Dominiak BC, Fanson BG, Collins SR, Taylor PW. Automated locomotor activity monitoring as a quality control assay for mass-reared tephritid flies. Pest Manag Sci. 2014; 70(2):304–9.

33. Shuttleworth LA, Khan MAM, Collins D, Osborne T, Reynolds OL. Wild bacterial probiotics fed to larvae of mass-reared Queensland fruit fly [*Bactrocera tryoni* (Froggatt)] do not impact long-term survival, mate selection, or locomotor activity. Insect Science. 2019; 27(4):745-755.

34. Bertolini E, Kistenpfennig C, Menegazzi P, Keller A, Koukidou M, Helfrich-Förster C. The characterization of the circadian clock in the olive fly *Bactrocera oleae* (Diptera: Tephritidae) reveals a *Drosophila*-like organization. Sci Rep. 2018; 8(1):1-12.

35. Kyritsis GA, Augustinos AA, Livadaras I, Cáceres C, Bourtzis K, Papadopoulos NT. Medfly-Wolbachia symbiosis: Genotype x genotype interactions determine host's life history traits under mass rearing conditions. BMC Biotechnology. 2019; 19(2):1-15.

36. Diamantidis AD, Papadopoulos NT, Carey JR. Medfly populations differ in diel and age patterns of sexual signalling. Entomol Exp Appl. 2008; 128(3):389–97.

37. Papanastasiou SA, Carey JR, Papadopoulos NT. Effects of early-life protein starvation on longevity and sexual performance of male medfly. PLoS One. 2019; 14(7): e0219518.

38. Boller EF. Rhagoletis cerasi and *Ceratitis capitata*. In: Sing P, Moore RF, editor. Handbook of Insect Rearing. 2nd ed. Amsterdam: the Netherlands: Elsevier. 1985; pp. 135–144.

39. Economopoulos AP, Judt S. Artificial rearing of the Mediterranean fruit fly (Diptera: Tephritidae): Size of oviposition holes. J Econ Entomol. 1989; 82(2):668–74.

40. Carey JR, Liedo P, Müller HG, Wang JL, Vaupel JW. A simple graphical technique for displaying individual fertility data and cohort survival: Case study of 1000 mediterranean fruit fly females. Funct Ecol. 1998; 12(3):359–63.

41. Müller HG, Carey JR, Wu D, Liedo P, Vaupel JW. Reproductive potential predicts longevity of female Mediterranean fruitflies. Proc R Soc B Biol Sci. 2001; 268(1466):445–50.

42. Halekoh U, Højsgaard S, Yan J. The R package geepack for generalized estimating equations. J Stat Softw. 2006; 15(2):1–11.

43. Liu C, Haynes PR, Donelson NC, Aharon S, Griffith LC. Sleep in populations of *Drosophila melanogaster*. eNeuro. 2015; 2(4).

44. Carey JR. Host-specific demographic studies of the Mediterranean fruit fly *Ceratitis capitata*. Ecol Entomol. 1984; 9(3):261–70.

45. Müller HG., Wang JL., Capra WB., Liedo P., Carey JR. Early mortality surge in protein-deprived females causes reversal of sex differential of life expectancy in Mediterranean fruit flies. Proc Natl Acad Sci. 1997; 94(6):2762–5.

46. Plácido-Silva MDC, Silva Neto, A. M. D., Zucoloto FS, Joachim-Bravo IS. Effects of different protein concentrations on longevity and feeding behavior of two adult populations of *Ceratitis capitata* Wiedemann (Diptera: Tephritidae). Neotrop Entomol. 2006; 35:747–52.

47. Diamantidis AD, Papadopoulos NT, Nakas CT, Wu S, Müller HG, Carey JR. Life history evolution in a globally invading tephritid: patterns of survival and reproduction in medflies from six world regions. Biol J Linn Soc. 2009; 97(1):106–17.

48. Papadopoulos NT, Liedo P, Müller HG, Wang JL, Molleman F, Carey JR. Cost of reproduction in male medflies: The primacy of sexual courting in extreme longevity reduction. J Insect Physiol. 2010; 56(3):283–7.

49. Novoseltsev VN., Carey RJ., Novoseltseva JA., Papadopoulos NT., Blay S., Yashin AI. Systemic mechanisms of individual reproductive life history in female medflies. Mech Ageing Dev. 2004; 125(1):77–87.

50. Warburg MS., Yuval B. Circadian patterns of feeding and reproductive activities of Mediterranean fruit flies (Diptera: Tephritidae) on various hosts in Israel. Ann Entomol Soc Am. 1997; 90(4):487–95.

51. Yuval B., Kaspi ROY., Shloush S., Warburg MS. Nutritional reserves regulate male participation in Mediterranean fruit fly leks. Ecol Entomol. 1998; 23(2):211–5.

52. Ratliff EP, Mauntz RE, Kotzebue RW, Gonzalez A, Achal M, Barekat A, et al. Aging and autophagic function influences the progressive decline of adult *Drosophila behaviors*. PLoS One. 2015; 10(7):e0132768.

53. Catterson JH, Knowles-Barley S, James K, Heck MMS, Harmar AJ, Hartley PS. Dietary modulation of *Drosophila* sleep-wake behaviour. PLoS One. 2010; 5(8):e12062.

54. Bochicchio PA, Pérez MM, Quesada-Allué LA, Rabossi A. Poor geotaxis correlated with haematoporphyrin-induced peroxidation of brain lipids as a predictor of medfly longevity reduction. Entomol Exp Appl. 2017; 164(2):131–40.

55. Toroser D, Orr WC, Sohal RS. Carbonylation of mitochondrial proteins in *Drosophila melanogaster* during aging. Biochem Biophys Res Commun. 2007; 363(2):418–24.

56. Grotewiel MS, Martin I, Bhandari P, Cook-Wiens E. Functional senescence in *Drosophila melanogaster*. Ageing Res Rev. 2005; 4(3):372–97.

57. Hendrichs J., Katsoyannos BI., Papaj DR., Prokopy RJ. Sex differences in movement between natural feeding and mating sites and tradeoffs between food consumption, mating success and predator evasion in Mediterranean fruit flies (Diptera: Tephritidae). Oecologia. 1991; 86(2):223–31.

58. Díaz-Fleischer F., Aluja M. Behavior of tephritid flies: a historical perspective. In: In Fruit Flies (Tephritidae) CRC Press [Internet]. 2001. pp. 57–88.

59. Demirel N. Behavior paradigms in the mediterranean fruit fly, *Ceratitis capitata* (Weidemann). J Entomol. 2007; 4(2):129–35.

60. Manrakhan A, Lux SA. Diel and lifetime patterns of feeding and reproductive activities of three African fruit flies, *Ceratitis cosyra*, *C. fasciventris* and *C. capitata* (Diptera: Tephritidae), in semi-field cages of different spatial scales. African Entomol. 2009; 17(1):8–22.

61. Bayoumy MH, El-Metwally MM. Daily flight activity rhythms of the peach and Mediterranean fruit flies using sexual and olfactory attractants. Acta Phytopathol Entomol Hungarica. 2017; 52(2):227–43.

62. Goldshtein E, Gazit Y, Hetzroni A, Timar D, Rosenfeld L, Grinshpon Y, et al. Long-term automatic trap data reveal factors affecting diurnal flight patterns of the Mediterranean Fruit fly. J Appl Entomol. 2021; 145(5):427–39.

# 8. Supporting information

**S1 Table: Variables of the GEEs with significant effects on the activity of flies by the factors: Sex (males, females) and time period (night, morning, midday, evening) of days 2, 11, 21, 42, 81.**

# 9. Author Contribution

**Conceptualization**: Vasilis G. Rodovitis, Stella A. Papanastasiou, Nikos A. Koulousis, James R. Carey, Nikos T. Papadopoulos
**Data curation:** Vasilis G. Rodovitis, Stella A. Papanastasiou, Evmorfia P. Bataka
**Formal Analyses:** Vasilis G. Rodovitis, Evmorfia P. Bataka, Christos T. Nakas, Nikos T. Papadopoulos
**Investigation:** Vasilis G. Rodovitis
**Methodology:** Vasilis G. Rodovitis, Stella A. Papanastasiou, Nikos T. Papadopoulos, James R. Carey
**Resources:** Nikos T. Papadopoulos, James R. Carey
**Project administration:** Nikos T. Papadopoulos, James R. Carey
**Supervision:** Nikos T. Papadopoulos, James R. Carey
**Writing- original draft:** Vasilis G. Rodovitis, Stella A. Papanastasiou, Nikos T. Papadopoulso
**Writing- reviewing & editing:** Vasilis G. Rodovitis, Stella A. Papanastasiou, Nikos T. Papadopoulos, Nikos A. Koulousis, James R. Carey, Evmorfia P. Bataka, Christos T. Naka

Figure Click here to access/download;Figure;Fig1.tif

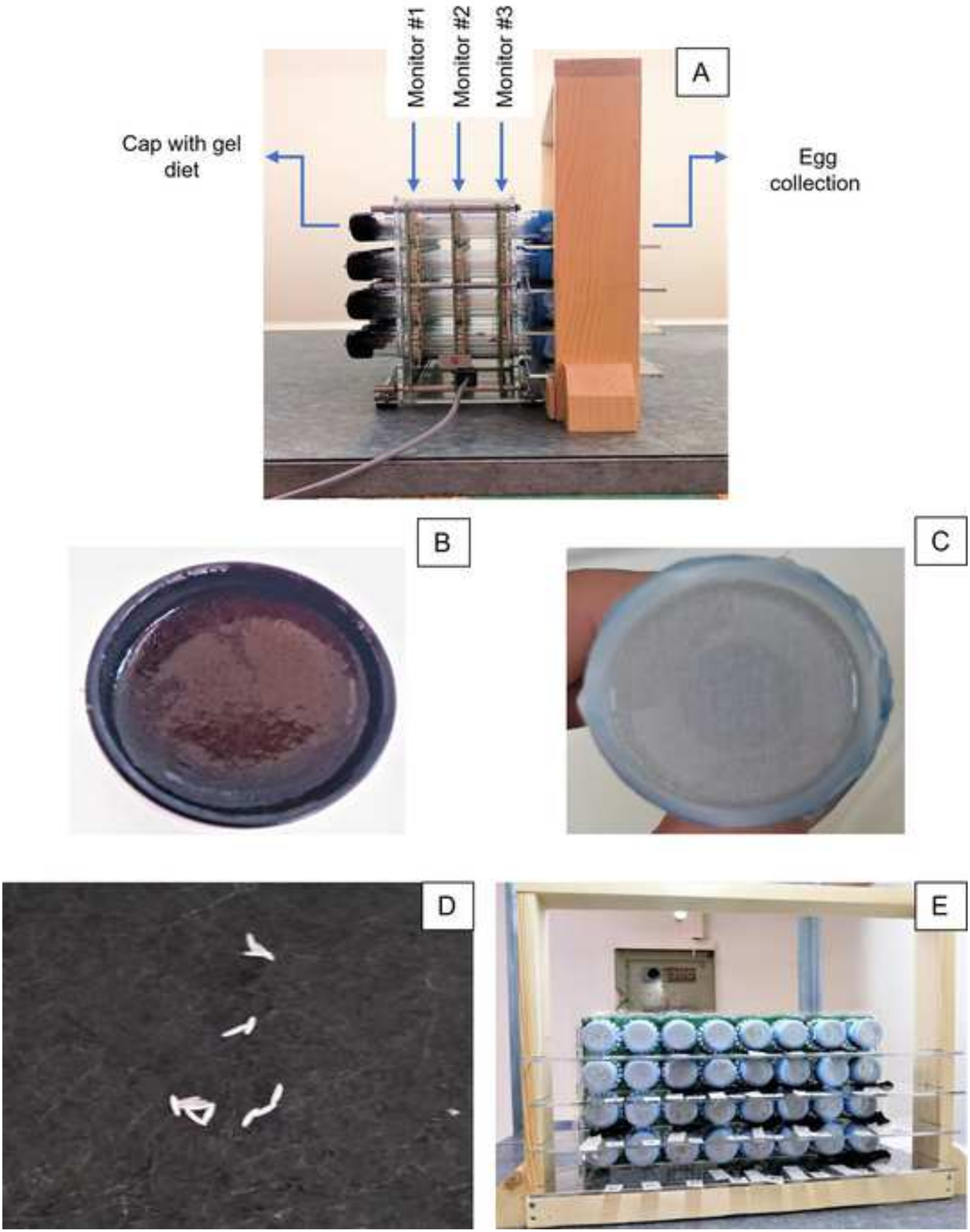

Figure
Click here to access/download;Figure;Fig2.tif

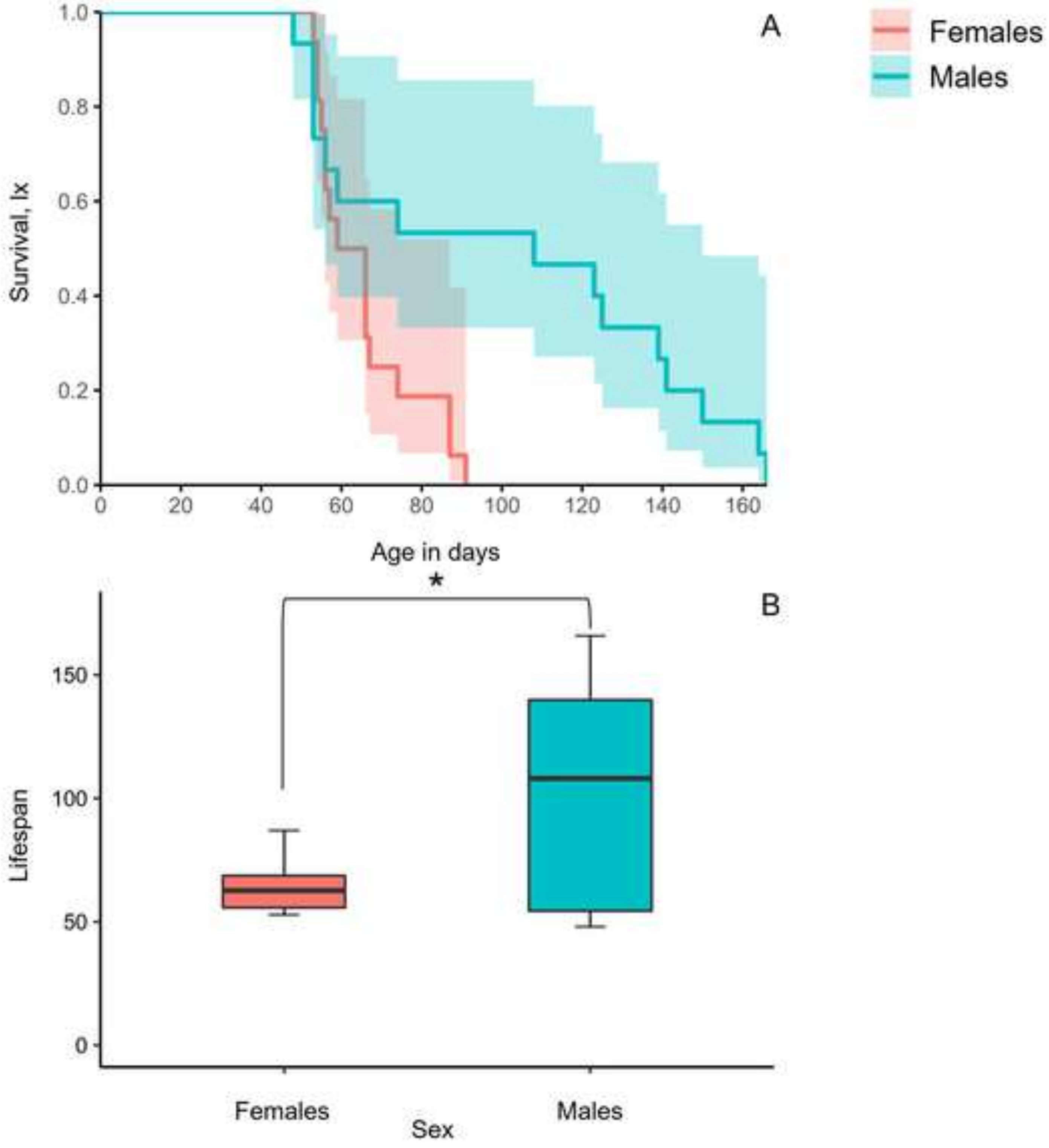

Click here to access/download;Figure;Fig3.tif

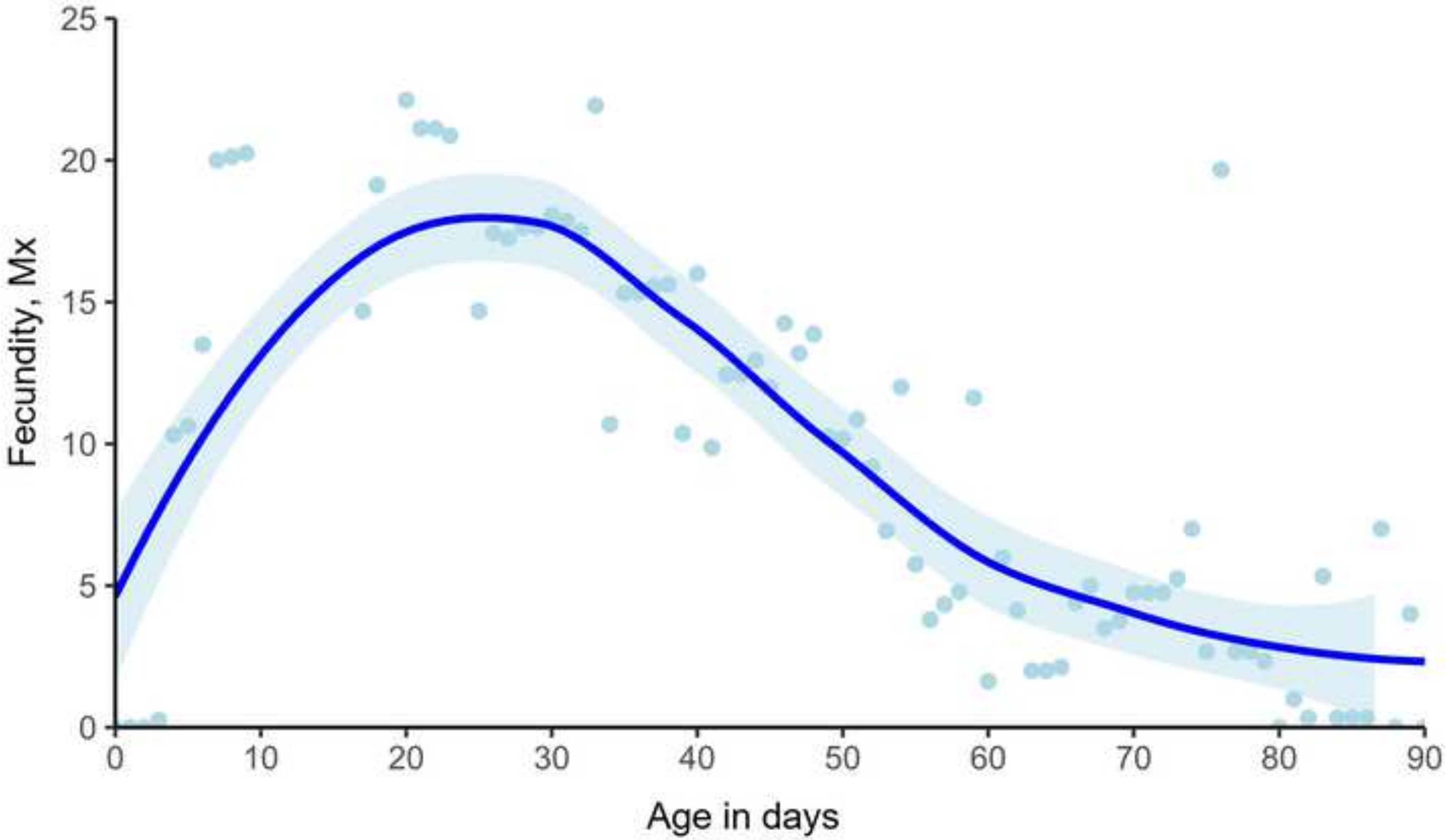

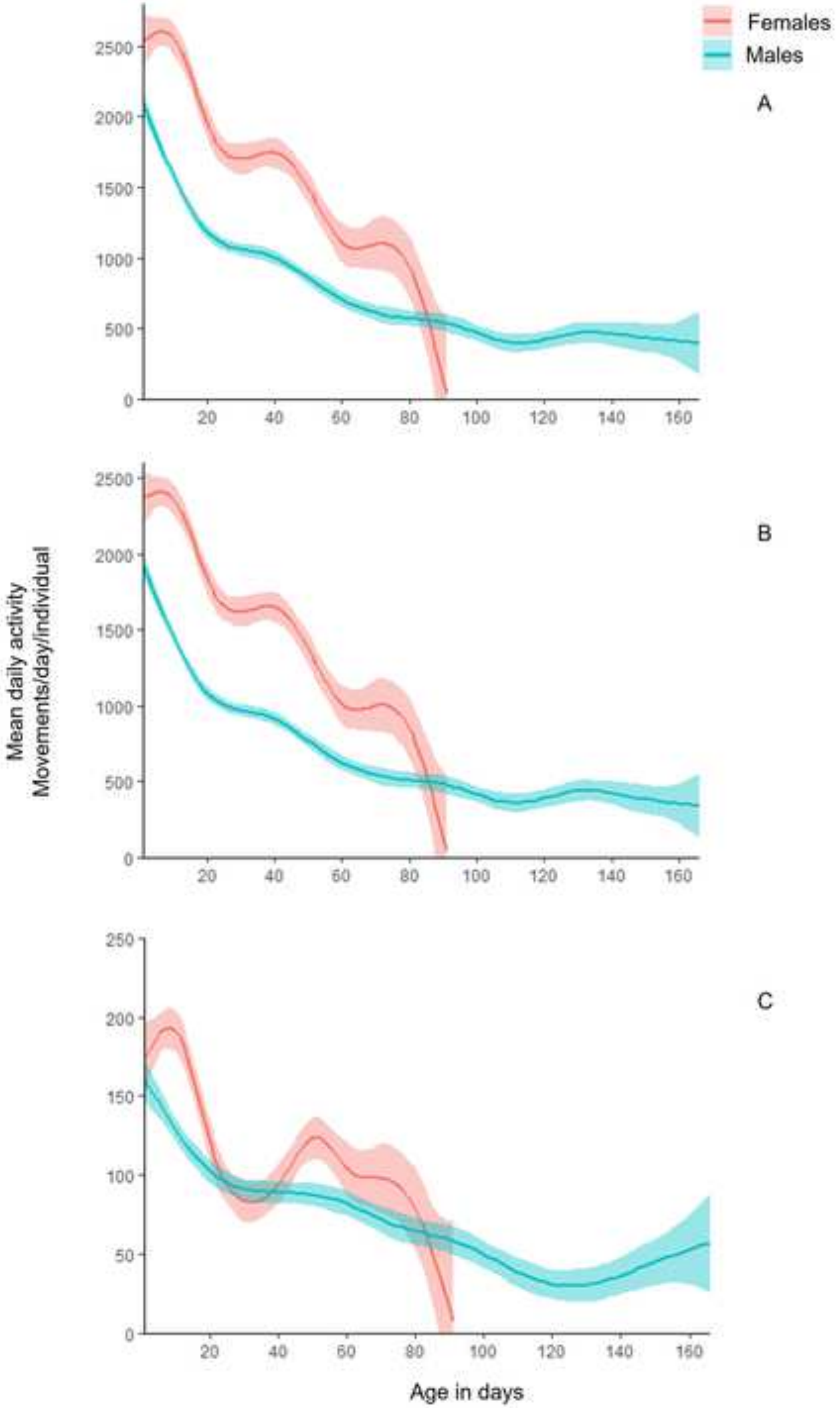
Females
Males
A
B
C
Mean daily activity
Movements/day/individual
Age in days
0
500
1000
1500
2000
2500
0
50
100
150
200
250
20
40
60
80
100
120
140
160

Figure Click here to access/download;Figure;Fig5.tif

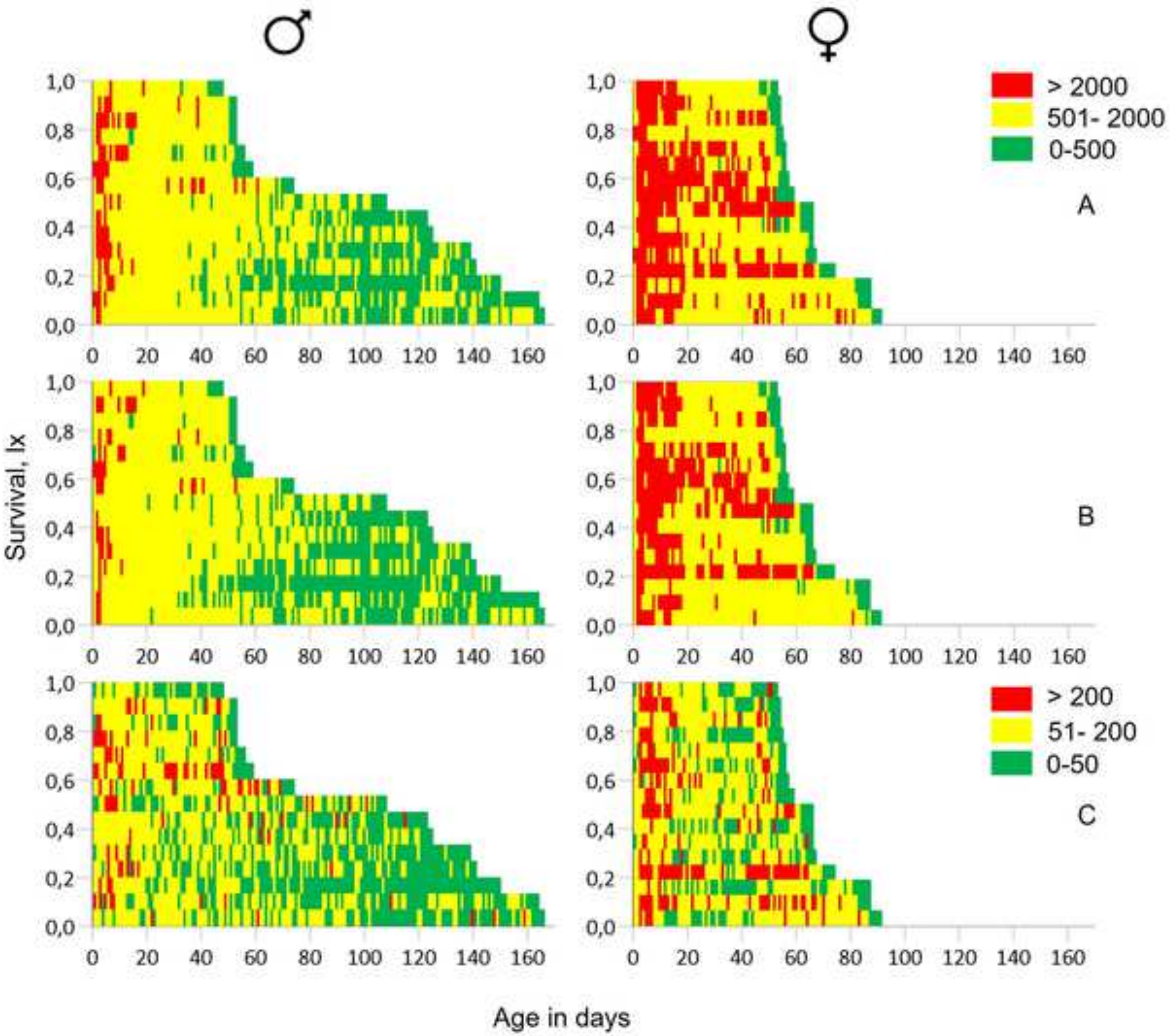

Figure

Click here to access/download;Figure;Fig6.tif

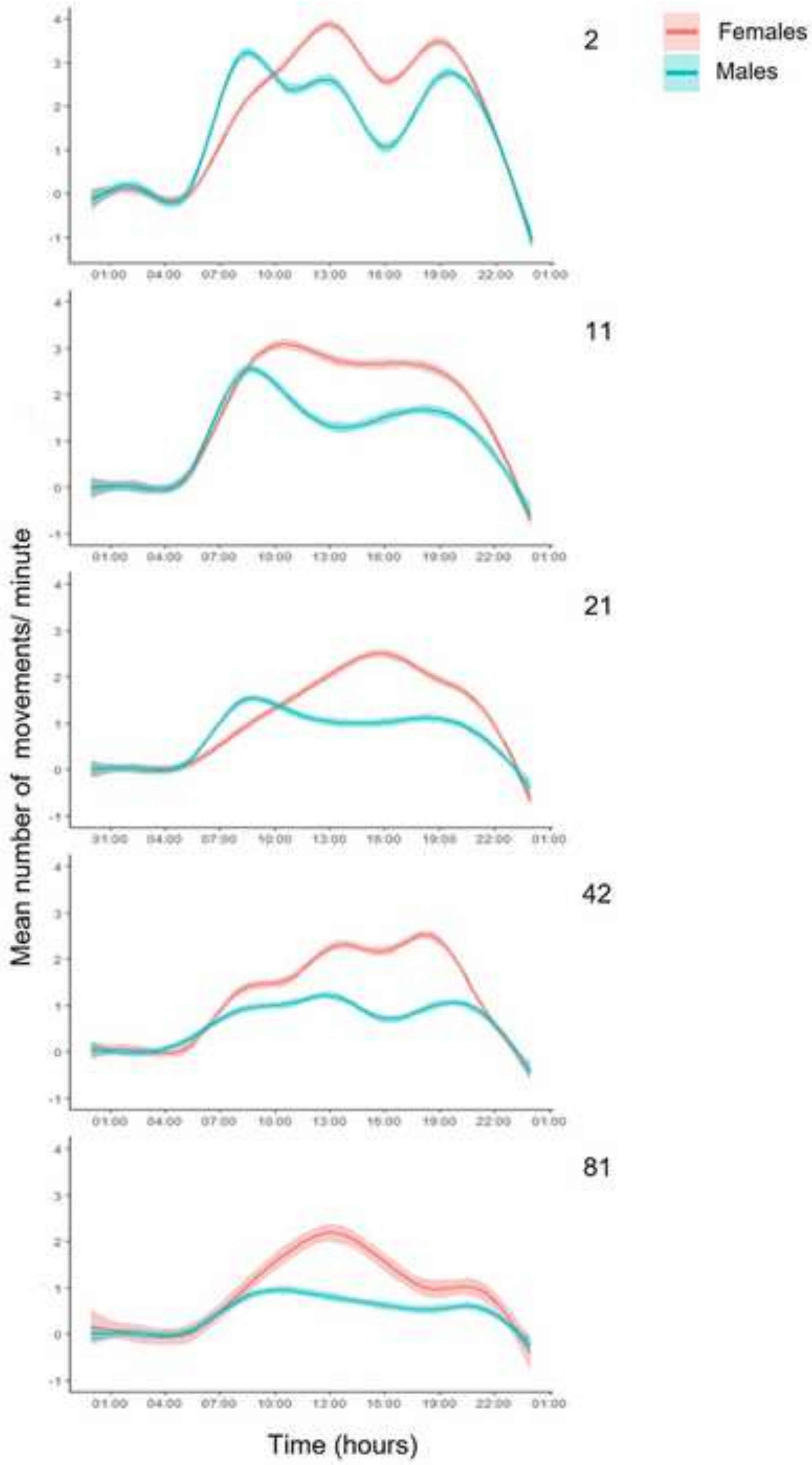

Click here to access/download
**Supporting Information**
Supporting Information.docx

Data Review URL

Click here to download Data Review URL
http://data.world/vasrod43/lam25system-medfly2021
